# Supplementary material for: Intestinal Farnesoid X Receptor Modulates Duodenal Surface Area but Does Not Control Glucose Absorption in Mice
Source: Int J Mol Sci. 2023 Feb 18;24(4):4132. doi: 10.3390/ijms24044132 (PMC9961586; doi:10.3390/ijms24044132)
Supplement: Supplementary file 1 [file ijms-24-04132-s001.zip › ijms-2147259-supplementary.pdf]

Intestinal Farnesoid X Receptor modulates duodenal surface area but does not control glucose absorption in mice

Jiufang Yang [1], Theo H van Dijk [1], Martijn Koehorst [2], Rick Havinga [1], Jan Freark de Boer [1], Folkert Kuipers [1,2,3,*] and Tim van Zutphen [1,4,*]

[1] Department of Pediatrics, University of Groningen, University Medical Center Groningen, Groningen, 9700RB, the Netherlands
[2] Department of Laboratory Medicine, University of Groningen, University Medical Center Groningen, Groningen, 9700RB, the Netherlands
[3] European Research Institute for the Biology of Ageing (ERIBA), University of Groningen, University Medical Center Groningen, 9700RB Groningen, the Netherlands
[4] Faculty Campus Fryslân, University of Groningen, 8911CE Leeuwarden, the Netherlands
* Correspondence: F Kuipers: f.kuipers@umcg.nl; T van Zutphen: t.van.zutphen@umcg.nl; Tel.: +31 58 288 2132

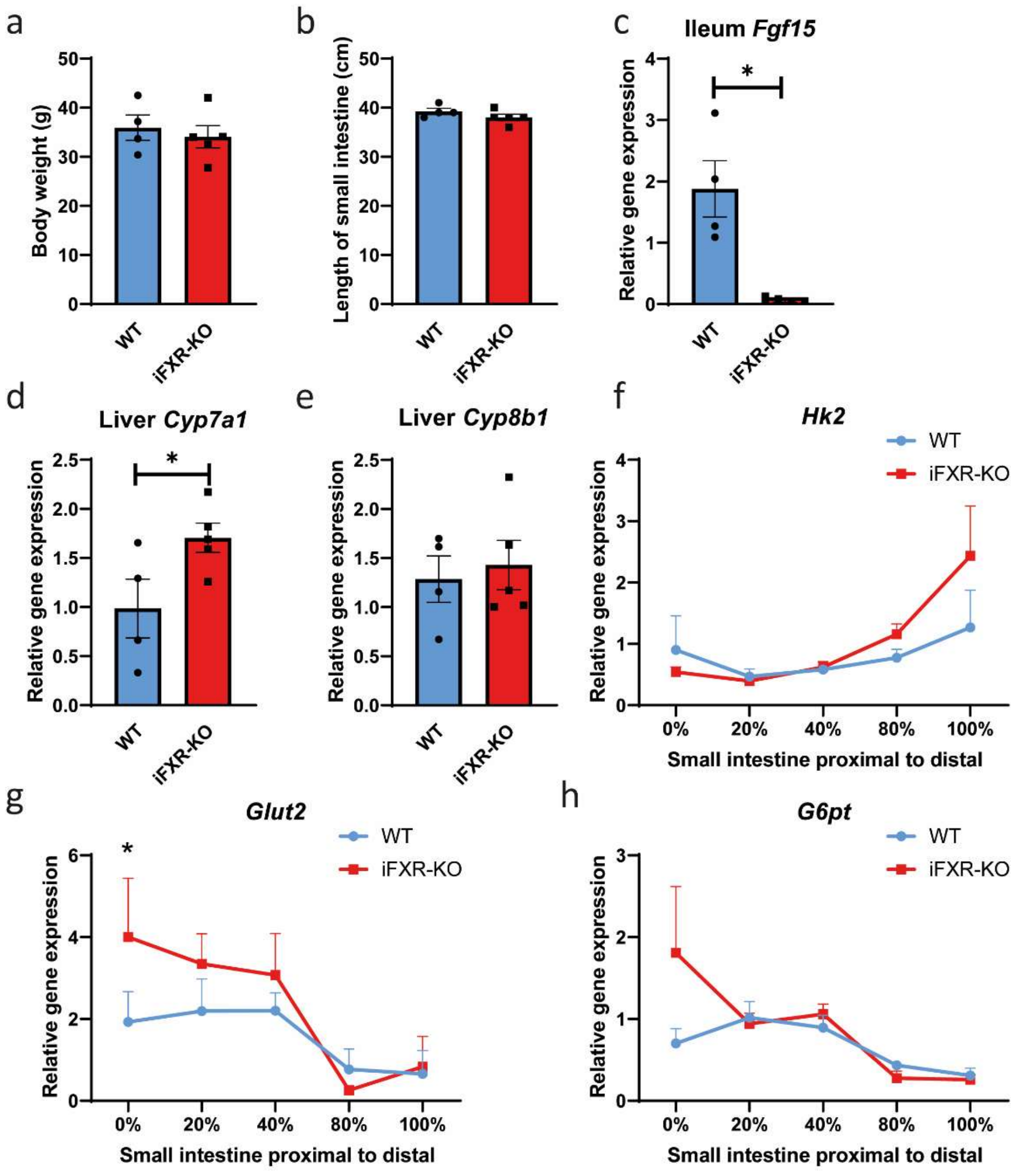

Figure S1. FXR deficiency in intestine does not lead to delayed glucose absorption appearance (**a**) Body weight and (**b**) intestine length of wild-type (WT) and intestine-specific FXR$^{-/-}$ (iFXR-KO) mice on a chow diet; (**c**) Quantitative real-time PCR of *Fgf*15 expression in ileum, (**d**) *Cyp7a1* and (**e**) *Cyp8b1* expression in liver of WT and iFXR-KO mice on a chow diet; (**f-h**) Quantitative real-time PCR of expression of glucose absorption relevant genes (*Hk2*, *Glut2* and *G6pt*) in five parts of intestine (0, 20, 40, 80 and 100% of small intestine from proximal to distal) from WT and iFXR-KO mice on chow diet. All panels: n=4-5/group, data are represented as mean ± SEM. **p*<0.05, when iFXR-KO mice are compared with WT mice.

A novel dual-label glucose kinetics approach

To avoid surgical interventions prior to experiments, such as those that require a permanent catheter in the jugular vein for continuous infusions of stable-isotope labeled glucose tracers, we designed a method with two labeled glucose tracers based on our previous non-invasive studies [70], i.e. one stable isotope labeled glucose tracer is administered intravenous (IV) and a second tracer orally (OR).

Complete absorption curves were compared to estimate the glucose absorption rate employing the "Wagner-Nelson method", a pharmacokinetic approach to estimate absorption over time [71]. The measured fractional contribution over time of both tracers were measured, after which the fractional absorption of the orally administered bolus can be calculated as the ratio of the both area under the concentration-time curve (AUC)'s corrected by the administered bolus sizes of both tracers. At any time point of the experiment, the total amount of an administered tracer appearance (A_{ap}) is equal to the amount in the body (A) and the amount eliminated from the body (A_{el}) until that time point, assuming that each tracer as well as natural abundant glucose has an equal elimination rate (**Equation 1**).

Total amount of an administered tracer appearance (A_{ap})	$A_{ap} = A + A_{el}$	Equation 1

Now, $A = C \cdot V$, $A_{el} = CL \cdot \int_0^t C \cdot dt$ and $CL = k_{el} \cdot V$ (C represents the concentration of labelled glucose, V represents apparent volume of distribution, CL represents total clearance of glucose from plasma, k_{el} represents rate constant in the elimination kinetics), which on substitution into Equation 1 gives **Equation 2**:

Total amount of an administered tracer appearance (A_{ap})	$A_{ap} = C \cdot V + k_{el} \cdot V \cdot \int_0^t C \cdot dt$	Equation 2

For IV-administered glucose tracer kinetics, IV-bolus concentrations obtained from GC-MS analysis (**Figure S2a**), the total amount of appearance during the whole experiment equals the bolus size (^{IV}B) at the end of the experiment its concentration is zero (^{IV}C=0) (**Equation 3** and **Equation 4**). The apparent volume of distribution can subsequently be calculated (**Equation 5**).

Total amount of intravenous administered bolus appearance ($^{IV}A_{ap}$)	$^{IV}A_{ap} = V(^{IV}C + k_{el} \cdot \int_0^t {^{IV}C} \cdot dt)$	Equation 3
Bolus size (^{IV}B)	$^{IV}B = k_{el} \cdot V \cdot {^{IV}AUC}$	Equation 4
Apparent volume of distribution (V)	$V = \frac{^{IV}B}{k_{el} \cdot {^{IV}AUC}}$	Equation 5

An example of estimation of cumulative amount of IV-administered glucose absorbed with time can be found in the table below:

IV-bolus									
Exp.time		C	ln(C)	C	AUC	k_e * AUC	C + k_e * AUC		
		measured		fitted curve	fitted curve		A_{ap}/ml	A_{ap}	Recovery
min		μmol.ml^{-1}		μmol.ml^{-1}	μmol.min.ml^{-1}	μmol.ml^{-1}	μmol.ml^{-1}	μmol.kg^{-1}	
0		0.00		2.464	0.0	0.00	0.00	0.0	0%
5		1.62	0.481	2.126	11.5	0.34	1.96	552.7	79%
15		1.47	0.384	1.583	30.0	0.89	2.35	664.7	95%
30	Time independent period	1.03	0.025	1.017	49.5	1.46	2.49	702.4	100%
45		0.66	-0.413	0.654	62.1	1.83	2.49	704.0	100%
60		0.41	-0.902	0.420	70.1	2.07	2.47	698.7	99%
90		0.18	-1.738	0.173	79.0	2.33	2.51	707.9	101%
time independent								703.2	100%
ln(C_0)			0.90						
C_0	μmol.ml^{-1}		2.46						
k_e	min^{-1}		-0.02948						
B	μmol.kg^{-1}							685	
V	ml.kg^{-1}						283		

The natural logarithm ln(C) was calculated in a fitted curve (**Figure S2b**) and the amounts of absorbed IV-administrated glucose and the recovery of IV-administered glucose bolus at different time points of test were obtained (**Figure S2c and S2d**). With respect to the OR-administered glucose tracer kinetics, the OR-bolus concentration was also obtained from GC-MS results (**Figure S2e**). Total amount of orally administered bolus appearance can be calculated by **Equation 6**. Since $A_{ap} = F \cdot B$, allowing fractional amount of appearance to be determined (**Equation 7** and **Equation 8**).

Total amount of orally administered bolus appearance ($^{OR}A_{ap}$)	$^{OR}A_{ap} = V(^{OR}C + k_{el} \cdot \int_0^t {^{OR}C} \cdot dt)$	Equation 6
Fractional amount of appearance ($^{OR}F_{ap}$)	$^{OR}F_{ap} = \frac{^{OR}A_{ap}}{^{OR}B}$	Equation 7
	$= V \cdot \frac{(^{OR}C + k_{el} \cdot \int_0^t {^{OR}C} \cdot dt)}{^{OR}B}$	Equation 8

An example of estimation of cumulative amount of OR-administered glucose absorbed with time can be found in the table below:

OR-bolus								
**Exp.time**	**C**	**ln(C)**	**C**	**AUC**	**k_e * AUC**	**C + k_e * AUC**		
	**measured**		**fitted curve**	**measured**		**A_{ap}/ml**	**A_{ap}**	**Recovery**
**min**	**μmol.ml^{-1}**		**μmol.ml^{-1}**	**μmol.min.ml^{-1}**	**μmol.ml^{-1}**	**μmol.ml^{-1}**	**μmol.kg^{-1}**	
**0**	0.00			0.0	0.00	0.00	0	0%
**5**	0.87			2.2	0.06	0.93	263	8%
**15**	1.99			16.4	0.48	2.47	698	21%
**30**	3.07			54.4	1.60	4.68	1322	40%
**45**	3.57			104.2	3.07	6.64	1876	57%
**60**	2.88			152.6	4.50	7.38	2086	64%
**90**	1.56			219.1	6.46	8.02	2265	69%
**k_e**	**min^{-1}**	-0.02948						
**B**	**μmol.kg^{-1}**						3283	
**V**	**ml.kg^{-1}**					283		

Subsequently the amounts of OR-administrated glucose bolus and the recovery of OR-administered glucose bolus at different time points of the experiment were calculated (**Figure S2f and S2g**). With respect to the total glucose appearance, total glucose concentration was measured from the tail-tip at indicated time points over 90 minutes using a hand-held device (**Figure S2h**). Finally, the total glucose appearance was calculated (**Figure S2i**) according to **Equation 9**:

Total glucose appearance ($^{Total}A_{ap}$)	$^{Total}A_{ap} = V(^{Total}C + k_{el} \cdot \int_0^t {^{Total}C} \cdot dt)$	Equation 9

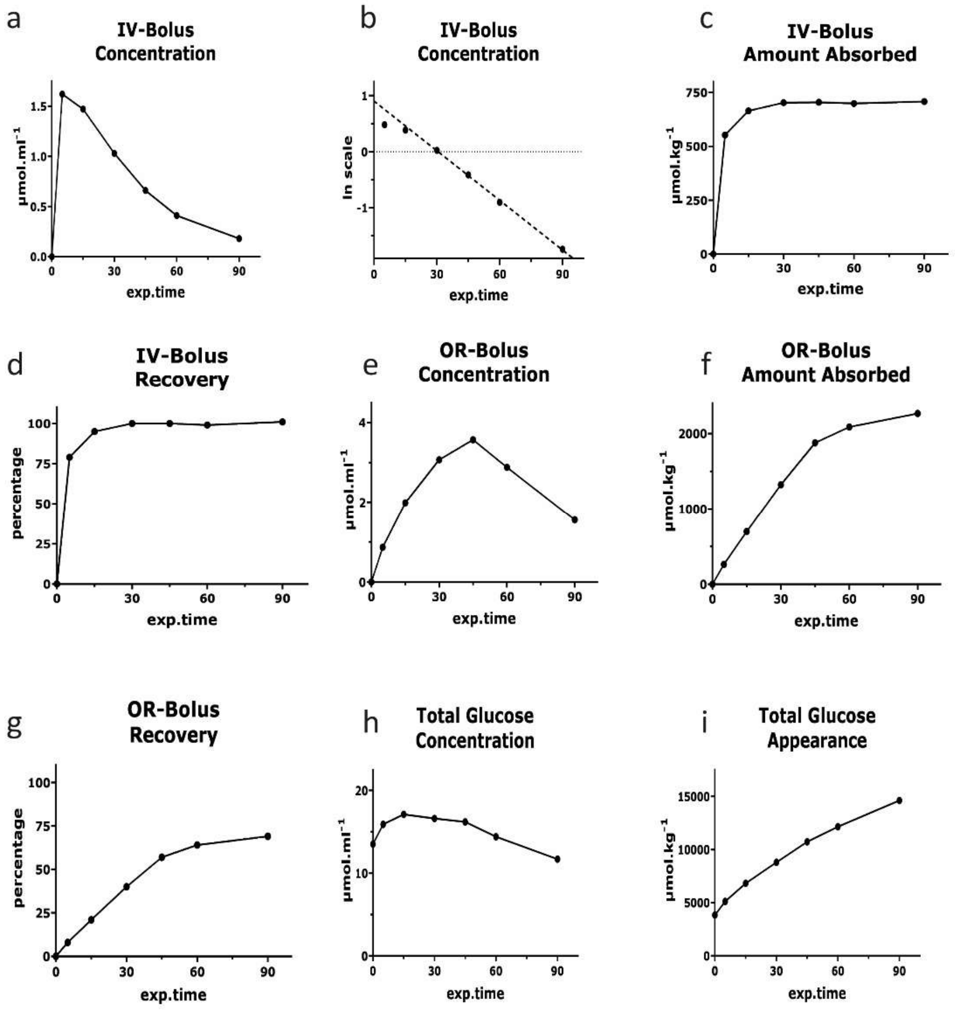

Figure S2. A novel dual-label glucose kinetics approach. (**a**) Intravenous (IV)-administrated glucose bolus concentration; (**b**) The natural logarithm of IV-administrated glucose bolus concentration; (**c**) The amounts of absorbed IV-administrated glucose bolus and (**d**) the recovery of IV-administrated glucose bolus during 90-min period of glucose absorption test; (**e**) Orally (OR)-administrated glucose bolus concentration; (**f**) The amounts of absorbed OR-administrated glucose bolus and (**g**) the recovery of OR-administrated glucose bolus during 90-min period of glucose absorption test; (**h**) Total glucose concentration and (**i**) total glucose appearance during 90-min period of glucose absorption test.

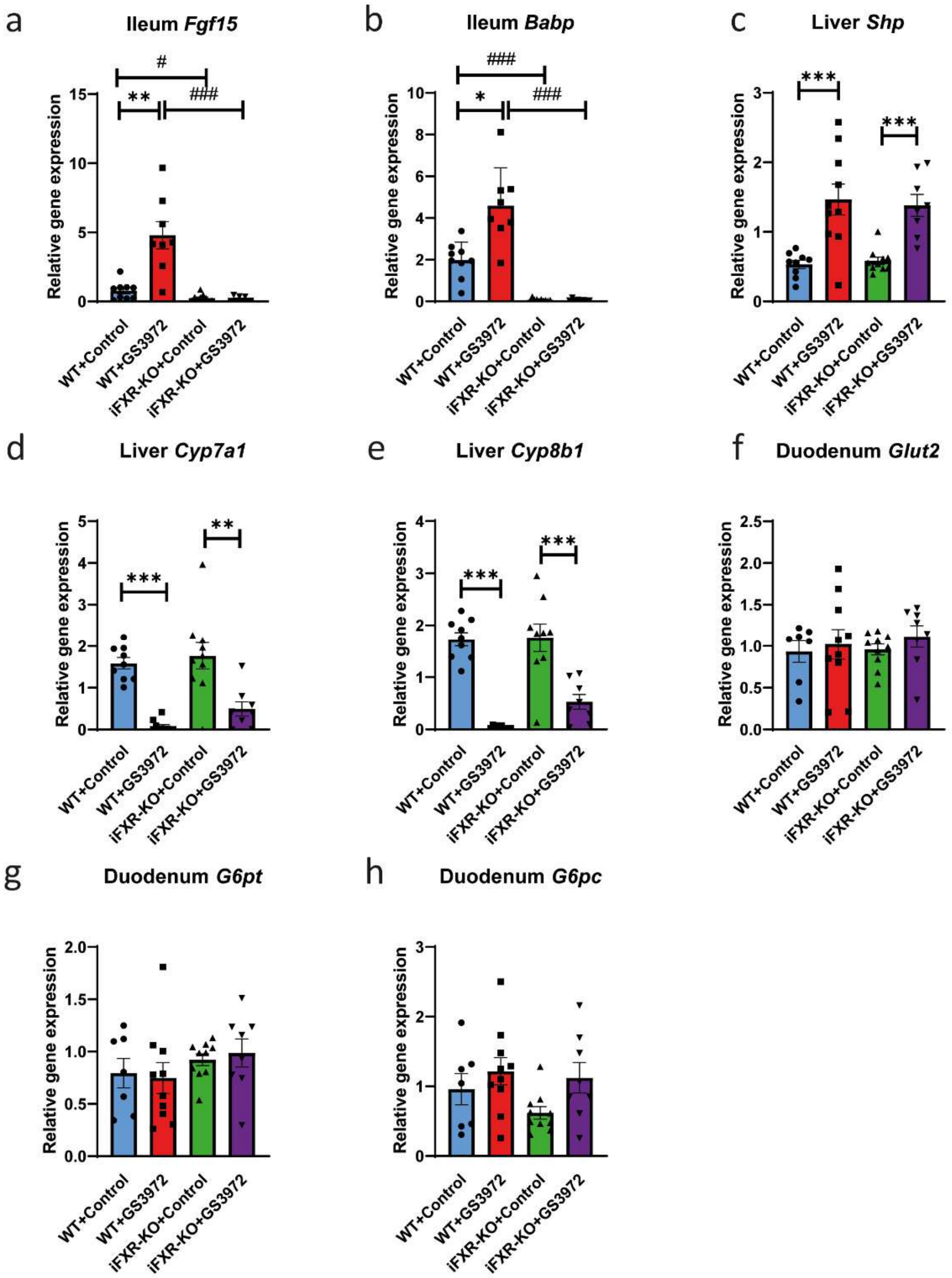

Figure S3. GS3972 activates FXR in liver but not in ileum of iFXR-KO mice. (**a-b**) Quantitative real-time PCR of FXR relevant gene expression in ileum (*Fgf15*, *iBabp*) and (**c-e**) in liver (*Shp*, *Cyp7a1* and *Cyp8b1*), and (**f-h**) glucose absorption gene expression (*Glut2*, *G6pt* and *G6pc*) of wild-type (WT) and intestine-specific $FXR^{-/-}$ (iFXR-KO) mice treated with or without GS3972 for one week under high-fat-diet (HFD) condition. Statistical significance was determined using Kruskal-Wallis H-test followed by post-hoc Conover pairwise comparisons. All panels: n=7-9/group. Data are represented as mean ± SEM, $^{*}p<0.05$, $^{**}p<0.01$, $^{***}p<0.001$ when mice with GS3972 treatment compared with control, $^{\#}p<0.05$, $^{\#\#\#}p<0.001$ when iFXR-KO mice are compared with WT mice.

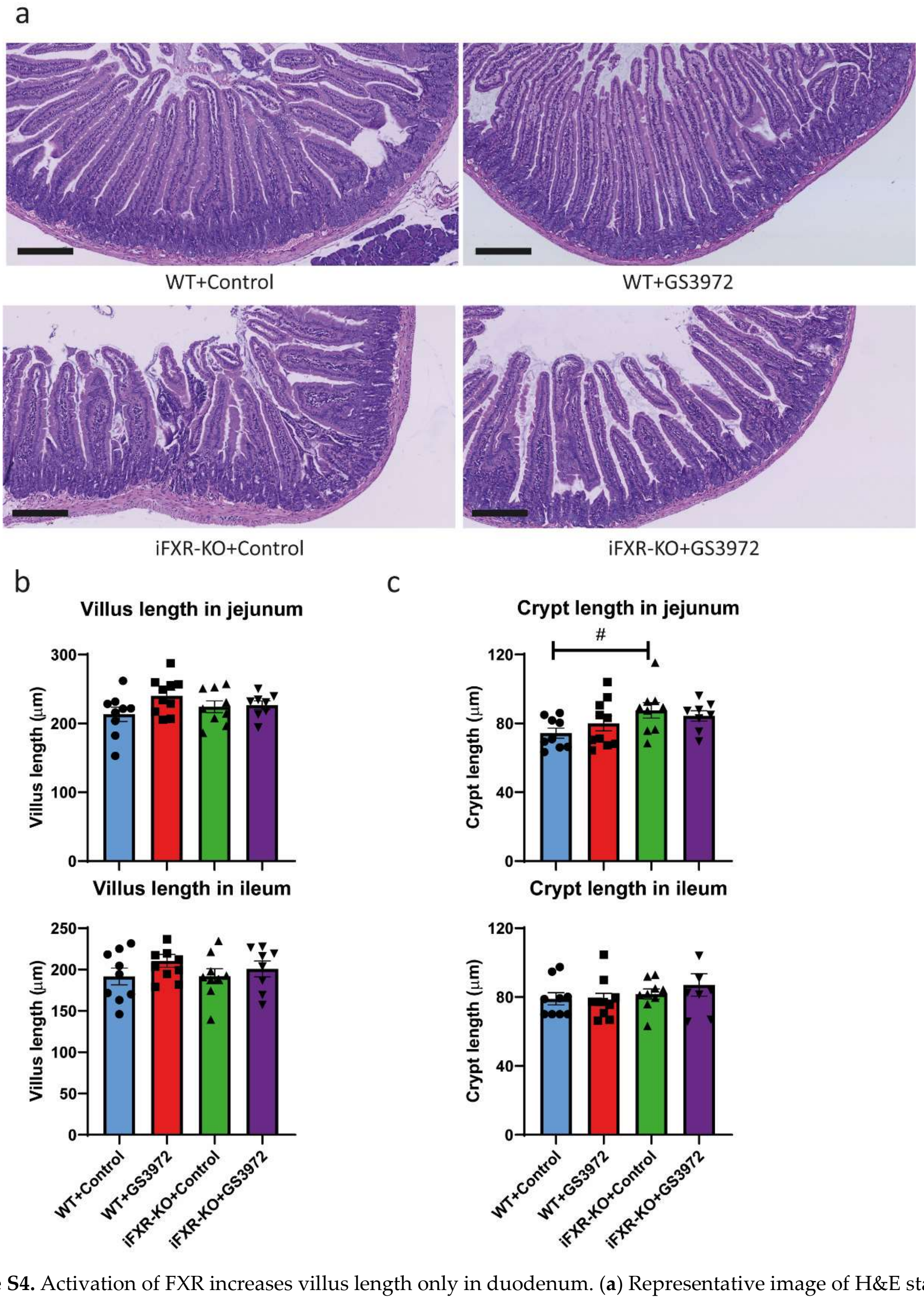

Figure S4. Activation of FXR increases villus length only in duodenum. (**a**) Representative image of H&E staining of duodenum from wild-type (WT) and intestine-specific $FXR^{-/-}$ (iFXR-KO) mice treated with or without GS3972 for one week under high-fat-diet (HFD) condition (magnification 10x; bar represents 200 μm); (**b**) Quantified analysis of villus length and (**c**) crypt length in jejunum and ileum from WT and iFXR-KO mice treated with or without GS3972 for one-week under HFD feeding (10 villi were selected from each mouse for qualification). Statistical significance was determined using Kruskal-Wallis H-test followed by post-hoc Conover pairwise comparisons. All panels: n=7-9/group. Data are represented as mean ± SEM, $^{\#}p<0.05$ when iFXR-KO mice compared with WT mice.

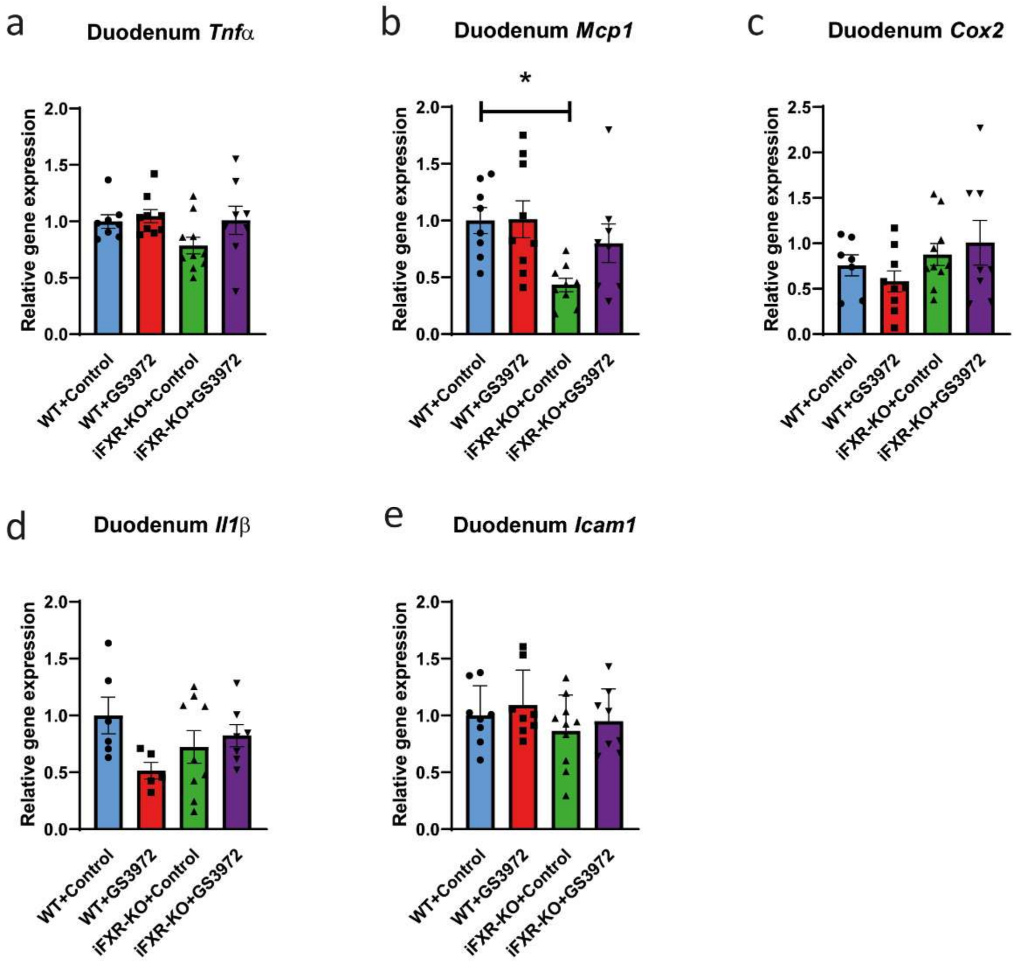

Figure S5. Activation of FXR does not affect the inflammatory gene expression in duodenum. (**a-e**) Quantitative real-time PCR of inflammatory gene expression (*Tnfα*, *Mcp1*, *Cox2*, *Il1β* and *Icam1*) in duodenum from wild-type (WT) and intestine-specific FXR$^{-/-}$ (iFXR-KO) mice treated with or without GS3972 for one week under high-fat-diet (HFD) conditions. Statistical significance was determined using Kruskal-Wallis H-test followed by post-hoc Conover pairwise comparisons. All panels: n=7-9/group. Data are represented as mean ± SEM, **p*<0.05 when mice with GS3972 treatment are compared with control, #*p*<0.05 when iFXR-KO mice are compared with WT mice.

Table S1 Primers used for qRT-PCR

Gene	Forward	Reverse
Fgf15	GCCATCAAGGACGTCAGCA	CTTCCTCCGAGTAGCGAATCAG<br>Probe: CGCTCATGCAGAGGTACCGCACG
Cyp7a1	CAGGGAGATGCTCTGTGTTCA	AGGCATACATCCCTTCCGTGA<br>Probe: TGCAAAACCTCCAATCTGTCATGAGACCTCC
Cyp8b1	AAGGCTGGCTTCCTGAGCTT	AACAGCTCATCGGCCTCATC<br>Probe: CGGCTACACCAAGGACAAGCAGCAAG
Hk1	CACCGGCAGATTGAGGAAAC	CTCAGCCCCATTTCCATCTCT<br>Probe: TCCCACTTCCGCCTCAGCAAGC
Hk2	GGAACCCAGCTGTTTGACCA	CAGGGGAACGAGAAGGTGAAA<br>Probe: TGCCTGGCCAACTTCATGGACAAGC
Sglt1	GTTGGAGTCTACGCAACAGCAA	GGGCTTCTGTGTCTATTTCAATTGT<br>Probe: TCCTCCTCTCCTGCATCCAGGTCG
G6pt	GAGGCCTTGTAGGAAGCATTG	CCATCCCAGCCATCATGAGTA<br>Probe: CTCTGTATGGGAACCCTCGCCACG
Glut2	AGAGGCATCGACTGAGCAGAA	AGGATGGGCTGTCGGTAATTG<br>Probe: TCTCCGTGATCCAGCTCTTCACGGA
G6pc	TTCCCTGTCACCTGTGAGACC	ACATAGTATACACCTGCTGCGCC<br>Probe: CAGGAAGTCCCTCTGGCCATGCC
I-babp	CCCCAACTATCACCAGACTTCG	ACATCCCCGATGGTGGAGAT<br>Probe: TCCACCAACTTGTCACCCACGACCT
Asbt	ACAGGCTGTAGTGGTGCTAATTATG	ACCAGAGAAATACCTGAGGTCCAT<br>Probe: CTGCCCTGGAGGAACTGGCTCCA
Shp	AAGGGCACGATCCTCTTCAA	CTGTTGCAGGTGTGCGATGT<br>Probe: ATGTGCCAGGCCTCCGTGCC
Tnfα	CCACCACGCTCTTCTGTCTA	CTGATGAGAGGGAGGCCATT<br>Probe: TGAACTTCGGGGTGATCGGTCCC
Mcp1	GGCTCAGCCAGATGCAGTTAA	AGCCTACTCATTGGGATCATCTT<br>Probe: CCCCACTCACCTGCTGCTACTCATTCA
Cox2	TTGTTGAGTCATTCACCAGACAGAT	GCCTTTGCCACTGCTTGTACA<br>Probe: CCCCAGCAACCCGGCCAGC
Il1β	ACCCTGCAGCTGGAGAGTGT	TTGACTTCTATCTTGTTGAAGACAAACC<br>Probe: CCCAAGCAATACCCAAAGAAGAAGATGGAA
Icam1	CGTGTGCCATGCCTTTAGCT	TCCAGTTATTTGAGAGTGGTACAGTACTG<br>Probe: CAGGTACACATTCCTGGTGACATTCCCATG

Table S2 Concentration of different bile acids from plasma and gallbladder in WT and iFXR-KO mice on 10-week HFD.

CA: cholic acid, (T)CDCA: (tauro)chenodeoxycholic acid, TUDCA: tauroursodeoxycholic acid, (A,B,O)MCA: (α,β,ω)muricholic acid, TMCA: tauromuricholic acid, TCA: taurocholic acid, DCA: deoxycholic acid. Analysis according to de Boer et al., 2020 [75].

**Bile acids in plasma**	**WT (μM mean ± SEM)**	**iFXR-KO (μM mean ± SEM)**
CA	0.026 ± 0.011	0.212 ± 0.066
TUDCA	0.053 ± 0.006	0.192 ± 0.096
TCA	0.240 ± 0.040	14.252 ± 7.008
CDCA	<0.05	0.041 ± 0.012
DCA	0.119 ± 0.011	0.288 ± 0.057
TCDCA	0.010 ± 0.005	0.043 ± 0.021
TDCA	0.114 ± 0.020	0.368 ± 0.132
A-MCA	0.011 ± 0.003	0.036 ± 0.007
B-MCA	0.056 ± 0.004	0.232 ± 0.067
O-MCA	0.119 ± 0.005	0.482 ± 0.180
TA-MCA	0.047 ± 0.004	0.336 ± 0.161
TB-MCA	0.086 ± 0.009	8.385 ± 4.169
TO-MCA	0.126 ± 0.010	3.968 ± 1.955

**Bile acids in gallbladder**	**WT (μM mean ± SEM)**	**iFXR-KO (μM mean ± SEM)**
CA	0.044 ± 0.007	0.165 ± 0.031
TUDCA	5.062 ± 1.361	3.529 ± 0.749
TCA	94.841 ± 24.419	101.091 ± 20.877
TCDCA	3.948 ± 1.123	3.055 ± 0.708
TDCA	6.008 ± 1.796	4.517 ± 0.892
A-MCA	0.042 ± 0.005	0.070 ± 0.010
B-MCA	0.098 ± 0.015	0.084 ± 0.016
O-MCA	0.101 ± 0.016	0.080 ± 0.012
TA-MCA	15.629 ± 4.322	12.437 ± 2.718
TB-MCA	48.564 ± 11.982	24.366 ± 5.637
TO-MCA	13.666 ± 3.238	7.037 ± 1.508
